# Association of serum coiled-coil domain containing protein 11 levels with coronary artery disease

**DOI:** 10.3389/fcvm.2026.1818052

**Published:** 2026-06-15

**Authors:** Jie Gao, Hongping Wang, Wenjun Wang, WenYing Hu, PengYong Yan, Jun Lu, ZongJun Liu

**Affiliations:** 1 Department of Endocrinology, Putuo Hospital, Shanghai University of Traditional Chinese Medicine, Shanghai, China; 2 Department of Cardiology, Putuo Hospital, Shanghai University of Traditional Chinese Medicine, Shanghai, China

**Keywords:** biomarker, cardiovascular disease, coiled coil domain containing protein 11 (CCDC11), coronary artery disease, inflammation

## Abstract

**Objective:**

This study aimed to assess the association between circulating levels of coiled coil domain containing protein 11 (CCDC11) and coronary artery disease (CAD).

**Methods:**

A total of 266 patients diagnosed with CAD and 95 controls were enrolled in our center from January 2024 to December 2024. The CAD cohort was further stratified into three subgroups based on the number of affected coronary vessels: the single-vessel group (*n* = 88), the double-vessel group (*n* = 74) and the triple-vessel group (*n* = 104). Serum concentrations of CCDC11 were quantified using an ELISA kit. Spearman correlation analysis was employed to evaluate the relationships between CCDC11 levels and other clinical parameters. Binary logistic regression analysis was conducted to determine the independent association of CCDC11 with CAD. The discriminative ability of CCDC11 for CAD was assessed using receiver operating characteristic (ROC) curve analysis.

**Results:**

Median serum CCDC11 amounts were significantly elevated in the CAD group compared to controls (20.4 vs. 13.8 ng/mL, *P* < 0.001). Furthermore, serum CCDC11 levels demonstrated a positive trend correlating with the number of lesioned coronary vessels (*P* for trend < 0.001). Logistic regression analysis revealed that CCDC11 was independently correlated with CAD. In ROC analysis, the area under the ROC curve (AUC) for CCDC11 in discriminating CAD was moderate but significantly higher than that for CRP (0.71 vs. 0.56, *P* < 0.01). Utilizing the maximum Youden index, an optimal CCDC11 cutoff value of 19.37 ng/mL was identified, yielding a sensitivity of 53.4% and specificity of 91.6% for CAD discrimination.

**Conclusions:**

Elevated serum levels of CCDC11 were positively associated with both the presence and severity of CAD, suggesting that CCDC11 may serve as a novel and clinically valuable biomarker for CAD.

## Introduction

Coronary artery disease (CAD) represents one of the most prevalent cardiovascular disorders globally, characterized by significant rates of mortality and morbidity (1, 2). Atherosclerosis constitutes a principal pathogenic mechanism underlying CAD and contributes to an elevated risk of adverse cardiovascular events, including stroke, myocardial infarction (MI), cardiac arrest, and even death (3). Atherosclerosis is typified by lipid accumulation within arterial walls, with chronic low-grade inflammation playing a critical role in its pathogenesis (4, 5). This condition is closely associated with dyslipidemia, endothelial dysfunction, and inflammatory processes (4, 5). Elevated levels of pro-inflammatory cytokines, such as interleukin-6 (IL-6), tumor necrosis factor-alpha (TNF-α), and interleukin-1β (IL-1β), have been implicated in the initiation and progression of CAD.

Coiled coil domain containing (CCDC) proteins, typically comprising approximately 200 amino acids, feature a highly conserved superhelical structural motif known as the coiled-coil domain. This domain consists of one or more α-helical peptides that intertwine to form a superhelical configuration. It has been estimated that roughly 10% of an organism's proteome includes sequences capable of forming coiled-coil structures (6). CCDC proteins participate in a variety of biological and pathological processes, exhibiting diverse functional roles depending on the specific tissue or cell type in which they are expressed (7). Previous studies have demonstrated that certain members of the CCDC family are implicated in cellular migration, proliferation, inflammation, and apoptosis (8–14). Among these, coiled-coil domain-containing protein 11 (CCDC11) is a member characterized by 514 amino acids and an estimated molecular weight of approximately 62 kDa (15). To date, research on CCDC11 remains limited, and its relationship with inflammatory processes is not well understood. Although CCDC11 is primarily known for its role in ciliary function and left-right patterning, emerging evidence suggests that primary cilia signaling can modulate key inflammatory pathways such as NF-κB and MAPK (16–18). Given that endothelial inflammation and dysfunction are central to the pathogenesis of atherosclerosis, we hypothesize that CCDC11 may contribute to CAD development through regulation of endothelial inflammation or pro-inflammatory cytokine release. Consequently, investigating the association between CCDC11 and cardiovascular diseases, including inflammation and atherosclerosis, holds significant clinical relevance. In the present study, we analyzed clinical data from patients with coronary artery disease (CAD) at our institution to evaluate the association between CCDC11 and CAD.

## Methods

### Study population and methods

Participants were recruited from inpatients who underwent coronary angiography at Putuo Hospital between January 2024 and December 2024. Coronary artery disease (CAD) was diagnosed by an experienced cardiologist based on angiographic findings. CAD was defined as the presence of stenosis exceeding 50% in at least one major coronary artery, including the left main coronary artery, left anterior descending artery, circumflex branch, or right coronary artery. Stenosis of 50% or greater in a single vessel was classified as single-vessel disease; stenosis of 50% or greater in two vessels was classified as double-vessel disease; and stenosis of 50% or greater in more than two vessels was classified as triple-vessel disease. Control subjects were those exhibiting less than 30% stenosis in all coronary arteries. Exclusion criteria included acute myocardial infarction, valvular heart disease, congestive heart failure, thrombotic disorders, severe hepatic impairment (defined as alanine transaminase or aspartate aminotransferase levels exceeding three times the upper limit of the normal reference range), severe renal dysfunction (glomerular filtration rate below 30 mL/min/1.73 m^2^), acute or chronic infections, and malignancies. Ultimately, a total of 361 patients were enrolled and categorized into two groups: the control group (*n* = 95) and the CAD group (*n* = 266). The CAD group was further subdivided based on the number of affected coronary vessels into the single-vessel (*n* = 88), the double-vessel (*n* = 74), and the triple-vessel (*n* = 104) disease subgroups. The study was carried out in compliance with the Declaration of Helsinki and obtained approval from the Institutional Review Board of the Ethics Committee at Shanghai University of Traditional Chinese Medicine Affiliated Putuo Hospital. Written informed consent was obtained from all participants prior to inclusion in the study.

### Anthropometric and biochemical measurements

Anthropometric measurements and biochemical parameters were obtained from all participants upon admission. Systolic blood pressure (SBP) and diastolic blood pressure (DBP) were each measured twice using a standard mercury sphygmomanometer, and the mean values were calculated. Height and weight were recorded following standardized procedures. Body mass index (BMI) was computed using the conventional formula: weight (kg) divided by height squared (m^2^). Venous blood samples were collected from all subjects after an overnight fast. Biochemical indices, including blood glucose, lipid profile, creatinine (Cr), and C-reactive protein (CRP), were analyzed utilizing a Beckman Coulter AU5800 automated biochemical analyzer. Glycated hemoglobin (HbA1c) concentrations were quantified utilizing the Tosoh Automated Glycohemoglobin Analyzer HLC-723G11. The estimated glomerular filtration rate (eGFR) was computed based on the CKD-EPI equation (19). Serum levels of CCDC11 were assessed concurrently with coronary imaging through a specific enzyme-linked immunosorbent assay (ELISA) kit (United States Biological, USA).

### Statistical analysis

Categorical variables were expressed as frequencies and percentages and analyzed using the Chi-square test. The normality of continuous variables was assessed via the Kolmogorov–Smirnov test. Variables exhibiting a normal distribution were evaluated using either the Student's *t*-test or one-way ANOVA and are reported as means with standard deviations (SD). Non-normally distributed variables were analyzed using the Mann–Whitney *U* test or Kruskal–Wallis test and are presented as medians with interquartile ranges (IQR). Spearman's correlation analysis was conducted to investigate the relationships between CCDC11 and various clinical parameters. Furthermore, logistic regression analysis was employed to examine the association between CCDC11 and coronary artery disease (CAD). Receiver operating characteristic (ROC) curve analysis was performed to assess the sensitivity and specificity of CCDC11 in discriminating CAD, with the optimal cut-off values determined based on the maximal Youden index. Areas under the ROC curves (AUCs) for CCDC11 and other clinical variables were calculated using MedCalc statistical software. Statistical significance was established at a two-tailed *P*-value below 0.05. All statistical analyses were performed using SPSS software, version 20.0.

## Results

### Clinical characteristics of the subjects

Clinical characteristics of all patients in CAD and control groups were displayed in Table 1. Compared with the control group, patients in the CAD group were older and showed a more frequent proportion of males and smokers. In addition, patients in the CAD group had significantly higher levels of SBP, TG, Cr, FBG, PBG, HbA1c, BNP than patients in the control group. The levels of HDL-C and eGFR were lower in the CAD group than those in the control group. There were no statistical differences in frequency of drinkers, BMI, DBP, TC, LDL-C, CRP, LVEF between the two groups (all *P* > 0.05). Interestingly, we found that CCDC11 levels in the CAD group were significantly increased compared with the control group (median 20.4 vs. 13.8, *P* < 0.001).

**Table 1:** Baseline clinical characteristics of all participants.

Clinical characteristic	All (*n* = 361)	Control (*n* = 95)	CAD (*n* = 266)	*P* value
Age (years)	62.0 (56.0–70.0)	57.0 (51.0–63.0)	64.0 (59.0–72.0)	**0** **.** **000**
Male (%)	237 (65.7%)	38 (40.0%)	199 (74.8%)	**0** **.** **000**
Smoking (%)	97 (26.9%)	15 (15.8%)	82 (30.8%)	**0** **.** **005**
Drinking (%)	53 (14.7%)	12 (12.6%)	41 (15.4%)	0.613
BMI (kg/m^2^)	24.3 ± 3.2	24.1 ± 3.1	24.3 ± 3.3	0.505
SBP (mmHg)	135.7 ± 20.1	127.5 ± 17.2	138.7 ± 20.2	**0** **.** **000**
DBP (mmHg)	77.0 (69.0–82.0)	76.0 (69.0–83.0)	77.0 (69.0–82.0)	0.745
TC (mmol/L)	4.2 (3.6–4.9)	4.3 (3.6–4.9)	4.2 (3.5–4.9)	0.552
TG (mmol/L)	1.3 (1.0–1.8)	1.1 (1.0–1.6)	1.3 (1.0–1.8)	**0** **.** **036**
HDL-C (mmol/L)	1.1 (0.9–1.3)	1.2 (1.0–1.4)	1.1 (0.9–1.3)	**0** **.** **001**
LDL-C (mmol/L)	2.5 (1.9–3.1)	2.6 (1.9–3.1)	2.5 (1.9–3.2)	0.726
eGFR (mL/1.73 m^2^/min)	75.6 (60.7–92.0)	84.4 (67.5–96.9)	71.3 (58.6–88.6)	**0** **.** **000**
Cr (mmol/L)	81.0 (71.0–90.0)	74.0 (62.0–82.0)	83.5 (74.8–92.0)	**0** **.** **000**
CRP (mg/L)	0.9 (0.4–2.8)	0.7 (0.4–2.2)	1.0 (0.4–3.0)	0.135
FBG (mmol/L)	4.9 (4.6–5.3)	4.7 (4.5–5.1)	5.0 (4.6–5.4)	**0** **.** **002**
PBG (mmol/L)	6.7 (5.7–8.0)	6.0 (5.3–7.0)	6.9 (5.9–8.3)	**0** **.** **000**
HbA1c (%)	5.7 (5.5–6.1)	5.7 (5.4–5.9)	5.7 (5.5–6.1)	**0** **.** **047**
BNP (pg/mL)	88.0 (38.0–250.5)	59.1 (30.8–139.8)	103.1 (45.1–292.8)	**0** **.** **007**
LVEF (%)	67 (62–70)	67 (64–70)	66 (62–70)	0.231
CCDC11 (ng/mL)	17.9 (12.6–26.5)	13.8 (11.9–16.7)	20.4 (13.5–30.6)	**0** **.** **000**
Medications				
Aspirin (%)	292 (80.9%)	57 (60.0%)	235 (88.3%)	**0** **.** **000**
Statin use (%)	262 (72.6%)	30 (31.6%)	232 (87.2%)	**0** **.** **000**
Anti-diabetic	3 (0.8%)	–	3 (1.1%)	–

Data were expressed as mean ± standard deviation for normally distributed continuous variables, median (interquartile range) for abnormally distributed variables and number (%) for category variables. For comparisons between groups, the independent samples *t* test was applied for normally distributed continuous variables and Mann–Whitney *U* test was used for skewed data. For categorical data, *χ*^2^ test or Fisher's exact test were used to test the differences between groups. BMI, body mass index; SBP, systolic blood pressure; DBP, diastolic blood pressure; TC, total cholesterol; TG, triglyceride; HDL-C, high-density lipoprotein cholesterol; LDL-C, low-density lipoprotein cholesterol; eGFR, estimated Glomerular filtration rate; Cr, creatinine; CRP, C reactive protein; FBG, fasting blood glucose; PBG, postprandial blood glucose; HbA1c, hemoglobin A1c; BNP, brain natriuretic peptide; LVEF, left ventricular ejection fraction.

bold values indicate statistical significance.

**Figure 1:**
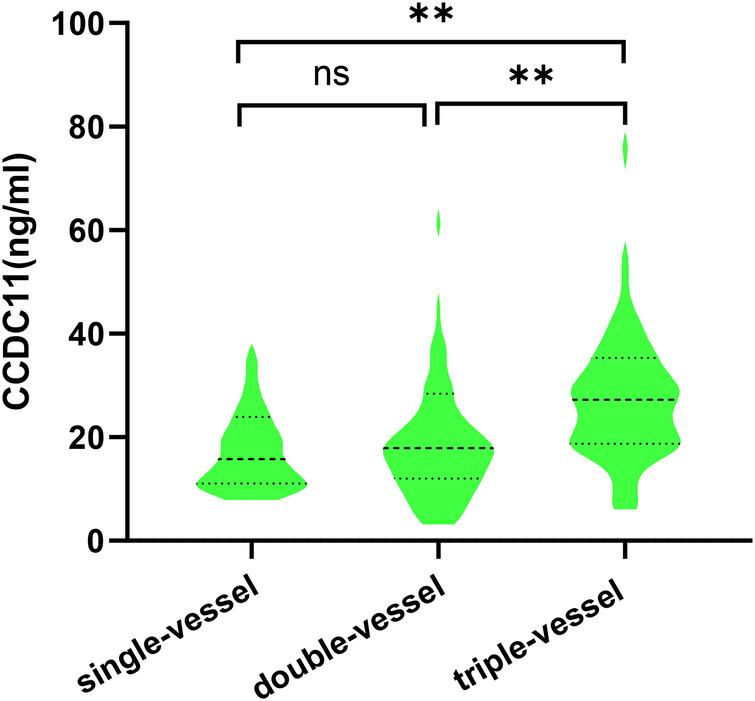
Levels of CCDC11 in patients with single-vessel, double-vessel and triple-vessel disease. **P < 0.01.

The clinical characteristics of the CAD subgroups were listed in Table 2. There were significant differences in the proportion of males, drinkers and age, HDL-C, BNP among these groups (all *P* < 0.05). CCDC11 levels in single-vessel, double-vessel and triple-vessel disease group were 15.8 (11.1–23.9), 17.9 (12.0–28.4), 27.2 (18.7–35.3) ng/mL, respectively. Circulating CCDC11 levels exhibited an increase corresponding to the number of affected coronary vessels (Figure 1). Notably, significant differences were observed between the single-vessel and triple-vessel groups, as well as between the double-vessel and triple-vessel disease groups (*P* < 0.001).

**Table 2:** Baseline clinical characteristics of CAD participants.

Clinical characteristic	Single vessel (*n* = 88)	Double vessel (*n* = 74)	Triple vessel (*n* = 104)	*P* value
Age (years)	61.0 (56.3–66.8)	65.0 (58.8–74.3)	66.0 (59.3–73.0)	**0** **.** **003**
Male (%)	56 (63.6%)	61 (82.4%)	82 (78.8%)	**0** **.** **011**
Smoking (%)	28 (31.8%)	25 (33.8%)	29 (27.9%)	0.682
Drinking (%)	22 (25%)	10 (13.5%)	9 (8.65%)	**0** **.** **007**
BMI (kg/m^2^)	24.0 ± 4.0	24.2 ± 2.6	24.7 ± 3.0	0.269
SBP (mmHg)	137.5 ± 21.8	139.7 ± 18.9	138.9 ± 19.9	0.749
DBP (mmHg)	78.0 (71.0–82.0)	76.5 (68.0–83.0)	77.0 (68.0–82.0)	0.728
TC (mmol/L)	4.2 (3.5–5.0)	4.3 (3.6–5.1)	4.1 (3.4–4.8)	0.591
TG (mmol/L)	1.2 (0.9–1.9)	1.3 (1.1–1.8)	1.5 (1.1–1.8)	0.264
HDL-C (mmol/L)	1.1 (1.0–1.3)	1.0 (0.9–1.2)	1.0 (0.8–1.3)	**0** **.** **009**
LDL-C (mmol/L)	2.6 (1.8–3.4)	2.5 (2.0–3.2)	2.4 (1.9–3.0)	0.417
eGFR (mL/1.73 m^2^/min)	73.3 (64.9–90.4)	71.8 (59.3–87.0)	69.1 (55.5–88.6)	0.363
Cr (mmol/L)	81.0 (73.0–87.8)	84.0 (73.0–91.3)	87.5 (77.0–98.0)	**0** **.** **012**
CRP (mg/L)	0.8 (0.4–3.6)	1.0 (0.4–3.1)	1.3 (0.5–2.6)	0.469
FBG (mmol/L)	5.0 (4.6–5.6)	4.9 (4.6–5.2)	5.1 (4.7–5.4)	0.177
PBG (mmol/L)	6.7 (5.7–8.1)	6.9 (5.8–8.4)	7.0 (6.2–8.4)	0.256
HbA1c (%)	5.7 (5.5–6.1)	5.7 (5.4–6.3)	5.8 (5.5–6.1)	0.698
BNP (pg/mL)	55.3 (29.0–173.7)	107.6 (50.1–313.3)	131.8 (65.5–423.8)	**0** **.** **001**
LVEF (%)	67.0 (63.8–70.0)	66.5 (62.0–70.0)	66.0 (60.0–69.0)	0.158
CCDC11 (ng/mL)	15.8 (11.1–23.9)	17.9 (12.0–28.4)	27.2 (18.7–35.3)	**0** **.** **000**
Medications				
Aspirin (%)	73 (83%)	70 (94.6%)	92 (88.5%)	0.071
Statin use (%)	77 (87.5%)	65 (87.8%)	90 (86.5%)	0.963
Anti-diabetic (%)	2 (2.3%)	-	1 (1%)	-

Data were expressed as mean ± standard deviation for normally distributed continuous variables, median (interquartile range) for abnormally distributed variables and number (%) for category variables. For comparisons among these groups, one-way ANOVA test was used for normally distributed continuous variables and Kruskal–Wallis test was used for skewed data. For categorical data, trend *χ*^2^ test was used to test the differences between groups. Abbreviations: as shown in Table 1.

bold values indicate statistical significance.

### Correlation between CCDC11 and clinical parameters

We assessed the relationship between plasma levels of CCDC11 and other clinical parameters in all subjects. Plasma CCDC11 levels were correlated positively with age, SBP, PBG, Cr and BNP in all subjects. In contrast, no significant correlation was observed between CCDC11 levels and any clinical parameters in control group and CAD group (Table 3).

**Table 3:** Correlation between plasma CCDC11 levels and clinical parameters.

Variables	All subjects (*n* = 361)		Control (*n* = 95)		CAD (*n* = 266)	
	*r*	*P*	*r*	*P*	*r*	*P*
Age	0.11	**0** **.** **030**	0.17	0.101	−0.03	0.614
SBP	0.13	**0** **.** **012**	0.01	0.935	0.05	0.403
DBP	0.02	0.779	−0.04	0.696	0.01	0.912
BMI	0.07	0.170	0.09	0.370	0.07	0.267
CRP	0.02	0.728	−0.14	0.221	0.04	0.551
eGFR	−0.06	0.270	0.01	0.955	0.01	0.822
Cr	0.11	**0** **.** **035**	−0.06	0.539	0.01	0.901
HbA1c	0.06	0.283	−0.14	0.217	0.05	0.462
FBG	0.08	0.136	−0.08	0.457	0.04	0.537
PBG	0.16	**0** **.** **006**	−0.12	0.292	0.12	0.073
TC	−0.08	0.141	−0.06	0.599	−0.07	0.259
TG	0.02	0.669	−0.03	0.797	0.01	0.970
HDL-C	−0.05	0.321	0.12	0.260	−0.03	0.687
LDL-C	−0.08	0.133	−0.14	0.167	−0.06	0.331
BNP	0.13	**0** **.** **032**	−0.01	0.912	0.11	0.116
LVEF	−0.08	0.129	−0.12	0.244	−0.05	0.452

Spearman correlation analysis was used to test the correlation of plasma CCDC11 and other clinical parameters in control and CAD group.

bold values indicate statistical significance.

### Association of CCDC11 levels with CAD

We analyzed the association between CCDC11 levels and CAD by different regression models. As shown in Table 4, total CCDC11 amounts were independently correlated with CAD in Model 1 (OR = 1.11, 95% CI 1.07–1.15), as well as adjusting for sex, age, BMI, SBP, smoking status, TG, HDL-C and HbA1c in Model 2 (OR = 1.11, 95% CI 1.06–1.16), and further adjustment for CRP, eGFR and medication use (aspirin and statin) in Model 3 (OR = 1.12, 95% CI 1.06–1.19).

**Table 4:** Association of CCDC11 levels with CAD.

CCDC11	Total	
	OR	95% CI
Model 1	1.11	1.07–1.15
Model 2	1.11	1.06–1.16
Model 3	1.15	1.08–1.23

Data were obtained using a binary logistic regression model. Model 1: unadjusted; Model 2: adjusted for sex, age, BMI, smoking status, SBP, TG, HDL-C and HbA1c; Model 3: Model 2 and additional adjustment for CRP, eGFR, medication use (aspirin and statin); CI, confidence interval; CAD, coronary artery disease.

### The discriminative ability of CCDC11 for CAD

**Figure 2:**
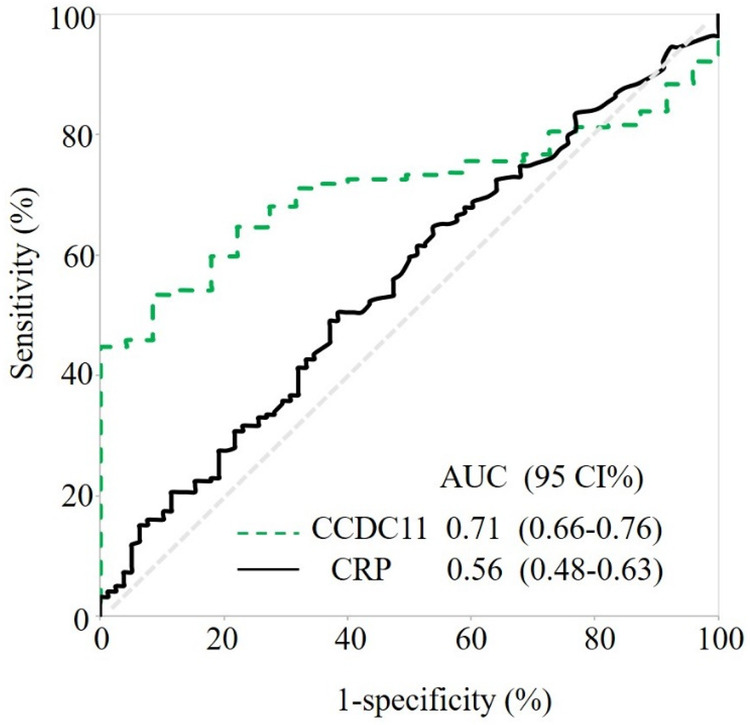
Receiver operating characteristic curves for CCDC11 in CAD patients. AUC, areas under the curve; CI, confidence interval.

**Figure 3:**
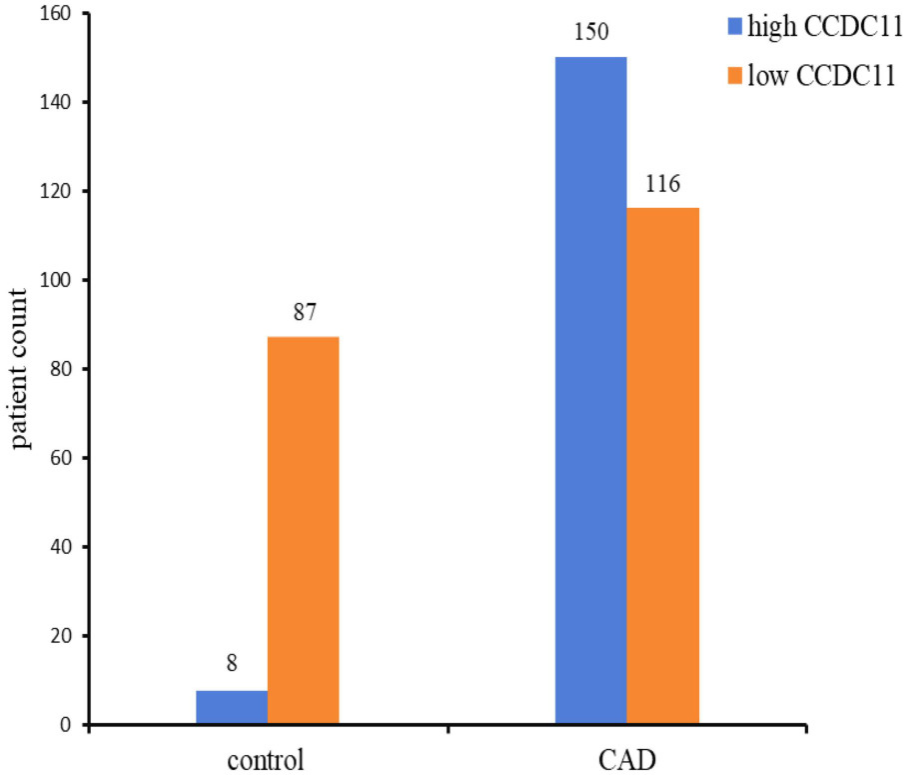
The numbers of patients with high CCDC11 levels between CAD and control groups. CCDC11 ≥ 19.37 ng/mL was defined as high CCDC11.

The ROC curve demonstrated that CCDC11 had a moderate discriminative ability for classifying CAD (AUC = 0.71, cutoff value = 19.37 ng/mL, sensitivity = 53.4%, specificity = 91.6%). Moreover, significant differences regarding the discriminative accuracy were observed between CCDC11 and CRP (AUC 0.71 vs. 0.56, *P* = 0.003) (Figure 2). When CCDC11 concentration was divided into dichotomous variable according to the cutoff (19.37 ng/mL), we found higher CCDC11 concentration was much more prevalent in CAD group (56.4% vs. 8.4%), and *χ*^2^ test also showed the test for trend reached a statistical significance (*P* < 0.001) (Figure 3).

## Discussion

CCDC proteins have been identified as being expressed across a broad spectrum of tissues and localized within various cell types, indicating their involvement in numerous critical physiological and pathological processes (20). For instance, CCDC7 may facilitate the proliferation of cervical cancer cells through the activation of interleukin-6 (IL-6) and vascular endothelial growth factor (VEGF) mediated by the JAK-STAT3 signaling pathway (21). CCDC3, a secretory protein that is highly conserved across species, is expressed in endothelial cells (ECs) and adipose tissues (22, 23). It functions as a negative regulator of tumor necrosis factor-alpha (TNF-α)-induced inflammatory responses in ECs by suppressing the activation of nuclear factor kappa B (NF-κB) (24). CCDC80 suppresses the phosphorylation of extracellular signal-regulated protein kinase 1/2 (ERK1/2) and reduces the expression of lipoprotein lipase (LPL) activity. This inhibition results in elevated serum triglyceride levels, thereby contributing to the progression of atherosclerosis (25–27). CCDC97 and CCDC107 have been identified as factors influencing the susceptibility to coronary artery disease (CAD) and the development of diabetes-related atherogenesis, respectively (28, 29). CCDC92 may influence serum lipid and glucose concentrations, thereby potentially elevating the risk of coronary artery disease (CAD) (30). Based on the aforementioned evidence, members of the CCDC family demonstrate a significant association with CAD.

CCDC11 comprises three predicted coiled-coil domains and is encoded by eight exons, resulting in a protein composed of 514 amino acids (15). CCDC11 was predominantly expressed in ciliated cells across various tissues and played a role in the induction of laterality as well as the motility of primary cilia (31, 32). The gene was initially characterized in a consanguineous family in which two siblings, both homozygous for a splice-site mutation, exhibited abnormalities in left–right patterning. This finding implicates CCDC11 in the processes of ciliogenesis or ciliary function (15, 33–36). Previous studies have demonstrated that the expression of CCDC11 is upregulated during the differentiation of multi-ciliated epidermal cells in Xenopus embryos (37), multi-ciliated tracheal epithelial cells in mice (38), and human airway epithelial cells (39).

In recent years, Perles et al. (35) identified mutations in the CCDC11 gene in individuals, which were linked to the occurrence of heart disease and laterality defects. Nonetheless, the association between CCDC11 and cardiovascular diseases, including atherosclerosis and coronary artery disease (CAD), has not been previously elucidated.

In our present study, we demonstrated that serum concentrations of CCDC11 were elevated in patients with CAD compared to control subjects, with levels exhibiting an increasing trend corresponding to the severity of CAD. Among CAD patients, serum CCDC11 levels were positively correlated with the number of affected coronary vessels. Moreover, binary logistic regression analysis identified CCDC11 as an associated factor for CAD. An interesting observation in our study was that serum CCDC11 levels correlated significantly with age, SBP, PBG, Cr, and BNP in the overall cohort. However, these correlations disappeared when the control and CAD groups were analyzed separately. The most plausible explanation was confounding by CAD status itself. Additionally, a ceiling effect within the CAD subgroup may contribute. Furthermore, the sample size in control group was relatively small, limiting statistical power to detect modest correlations, whereas the patients in CAD group showed characteristics such as older age, a higher proportion of males and smoking, which inherently reduced correlation coefficients even if a biological relationship exists. Importantly, the disappearance of these correlations upon stratification did not undermine the potential of CCDC11 as a biomarker for CAD. In contrast, it suggested that CCDC11 was more closely linked to the pathophysiological state of CAD than to individual demographic or metabolic variables, but future prospective investigations are warranted to ascertain whether alterations in CCDC11 can be independent of the status of CAD.

To the best of our knowledge, this investigation represents the first study to examine the association between CCDC11 and coronary artery disease (CAD). Notably, our findings indicate that CCDC11 serves as an independent correlate of both the prevalence and severity of CAD. Nonetheless, several limitations of the present study warrant consideration. First, considering the study was a cross-sectional study conducted in our single-center with a modest sample size, causal relationships between CCDC11 and CAD cannot be established. Second, due to significant baseline differences between the two groups, residual confounding cannot be excluded. Therefore, our findings should be considered hypothesis-generating rather than confirmatory. Third, the unusually low AUC for CRP suggested that our study population may not be representative of the general CAD population, which limits the generalizability of our findings.

## Conclusion

In summary, our findings indicate a correlation between elevated circulating concentrations of CCDC11 and increased odds of coronary artery disease (CAD), implying that CCDC11 may function as a biomarker for the presence and severity of CAD. Additional research is necessary to elucidate the precise role of CCDC11 in the pathogenesis of CAD.

## Data Availability

The original contributions presented in the study are included in the article/Supplementary Material, further inquiries can be directed to the corresponding authors.
